# Dyslipidemia in Chinese Pancreatic Cancer Patients: A Two-Center Retrospective Study

**DOI:** 10.7150/jca.60340

**Published:** 2021-07-03

**Authors:** Feiyang Wang, Li Huang, Jinyan Zhang, Junwei Fan, Heshui Wu, Junming Xu

**Affiliations:** 1 Department of General Surgery, Shanghai General Hospital, Shanghai Jiao Tong University School of Medicine, Shanghai, China.; 2 Department of Pancreatic Surgery, Wuhan Uniom Hospital, Huazhong University of Science and Technology, Wuhan, China.

**Keywords:** dyslipidemia, pancreatic cancer

## Abstract

**Background:** Pancreatic cancer (PC) is one of the most aggressive and lethal malignancies in the world. High cholesterol intake may have a certain association with an elevated risk of PC, though dyslipidemia in PC patients has rarely been reported. In this study, we compared serum lipids levels between PC and non-PC tumor patients and assessed their prognostic value in PC.

**Methods:** 271 patients treated at Wuhan Union Hospital from January 2012 to December 2016 and 204 individuals at Shanghai General Hospital from January 2018 to December 2019 were recruited. Their demographic parameters, laboratory data, pathological information, and clinical outcomes were extracted and analyzed. The mRNA expressions of related lipoprotein, low density lipoprotein receptor (LDLR) and high density lipoprotein binding protein (HDLBP), in PC tissues and paired noncancerous tissues and follow-up information were assessed based on the GEO database (GSE15471 and GSE62165) and TCGA database.

**Results:** A total of 172 non-PC tumor patients and 260 PC patients were finally eligible for our analysis. PC patients exhibited higher levels of serum triglyceride, cholesterol, and low-density lipoprotein (LDL) and a lower serum high-density lipoprotein (HDL) level on admission versus the non-PC tumor group. In PC patients, LDLR mRNA expression was upregulated, and HDLBP mRNA expression was downregulated in cancerous tissues compared to these levels in paired noncancerous tissues. The survival analysis revealed that dyslipidemia had a non-significant association with a poor prognosis, but PC patients with a high LDLR level were at risk of poor survival.

**Conclusion:** Dyslipidemia is detected in PC patients but has a non-significant relation to PC prognosis. However, LDLR may be a potential predictive marker for PC prognosis.

## Introduction

Pancreatic cancer (PC) is the 9^th^ most common malignant tumor worldwide. It has a dismal 5-year survival rate of less than 9%, which is expected to be the second deadliest cancer by 2030. Disappointingly, 80% of patients have developed regional or distant metastasis at first diagnosis 1.

With rapid advances in the diagnostic efficiency of imaging and laboratory techniques, there is an increased detection rate of PC over the last decades. But, some types of non-pancreatic cancer (non-PC) tumor, including pancreatic neuroendocrine tumor (PNET), intraductal papillary mucinous neoplasms (IPMNs), and pancreatic cystadenoma are still difficult to distinguish from PC without pathological means. Recently, research have found high cholesterol intake is associated with an elevated risk of PC 2-3, and lipid metabolism reprogramming is the key factor promoting PC development and chemotherapy resistance 4-5. Some lipoproteins, such as ApoE and ApoA-II, are recognized as potential biomarkers with higher sensitivity than current serum indicators for PC 2, 6. All these findings point directly at functional changes in lipid metabolism of PC. But by far, deviant serum lipid levels in PC and differences between PC and non-PC tumor patients have been rarely remarked.

In this work, we compared baseline serum lipid levels between PC versus non-PC tumor patients and examined their diagnostic utility in distinguishing PC from non-PC tumors. The prognostic potential of serum lipid levels in PC patients was also assessed. To better understand the role of lipid metabolism in the pathogenesis, we utilized GEO and TCGA data to identify differential targets between PC and non-PC tumors and evaluate their prognostic value in PC. This study provides fresh insights into lipid metabolism in PC development and progression.

## Materials and Methods

### Patients and data collection

**Figure 1:**
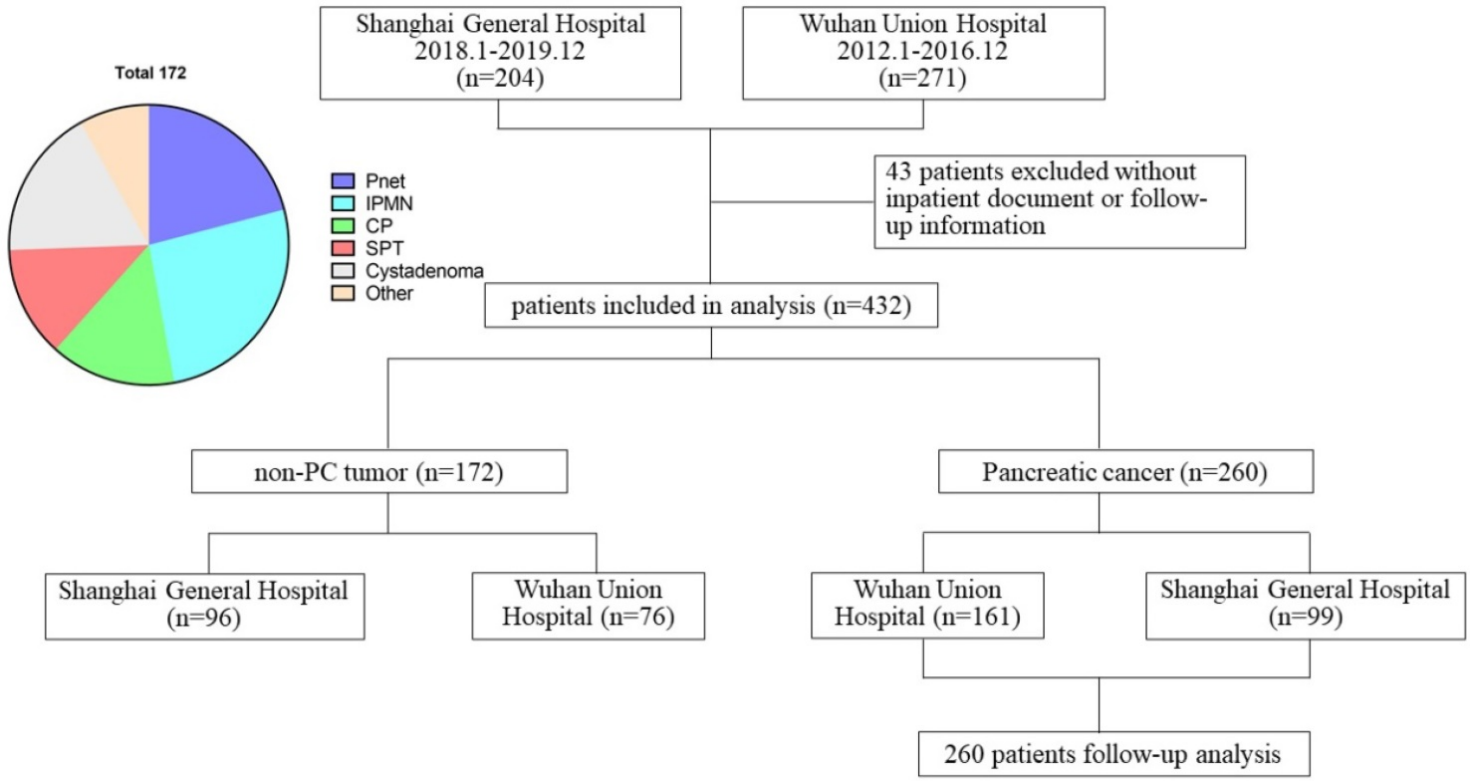
Schematic diagram regarding patient data.

We extracted the medical records of 271 patients hospitalized and undergoing surgical resection in Wuhan Union Hospital from January 2012 to December 2016 and 204 patients in Shanghai General Hospital from January 2018 to December 2019. Among them, 260 patients were pathologically confirmed as PC and 172 patients as non-PC tumor types (PNET, IPMN, chronic pancreatitis, solid pseudopapillary tumor, pancreatic cystadenoma, and other types). 43 patients without complete hospitalization or follow-up data were excluded (Figure 1). Demographic characteristics, laboratory results, serum lipids levels, surgical and pathological data were collected through electronic medical records and paper charts. Patient outcomes were evaluated based on outpatient records and telephone interviews. The follow-up was ended on March 7, 2020.

### Microarray data collection

Data sets of gene expressions used in this study were downloaded from the GEO database (http://www.ncbi.nlm.nih.gov/gds/) and derived from microarray studies comparing the mRNA expression profiling between PC and paired adjacent noncancerous tissues from patients. The mRNA expression data of GSE15471 (39 matched pairs of samples) and GSE62165 (118 PC tissue samples and 13 paracancerous tissue samples) were acquired to identify differential target genes of related lipoprotein in PC. Follow up information of PC patients were obtained from the Cancer Genome Atlas (TCGA) data portal (http://cancergenome.nih.gov/). According to the expression of related lipoprotein, a total of 177 PC patients with follow up information were selected for survival analysis.

### Statistical analysis

Statistical Production and Services Solution 19.0 (SPSS 19.0, SPSS Inc, Chicago, IL, USA) and GraphPad Prism 7 were used for statistical analysis. Continuous and categorical variables were presented as mean ± standard derivation (SD), as well as frequency and percentage, respectively. Normally distributed continuous variables were compared by using Student's *t*-test, and differences in categorical variables were analyzed using the chi-square or Fisher's exact test. The Mann-Whitney *U* test was used for comparisons in nonnormally distributed variables. A logistic regression analysis model was used to identify risk factors of PC. Only the variables with statistical significance in univariable analysis were included in further multivariable analysis. Odds ratios (ORs) and 95% confidence intervals (95% CIs) were presented. Patient overall survival (OS) was obtained using the Kaplan-Meier method. A P-value < 0.05 was considered a statistically significant difference.

## Results

### Clinical features, laboratory data, and serum lipids levels between PC versus non-PC tumors

**Figure 2:**
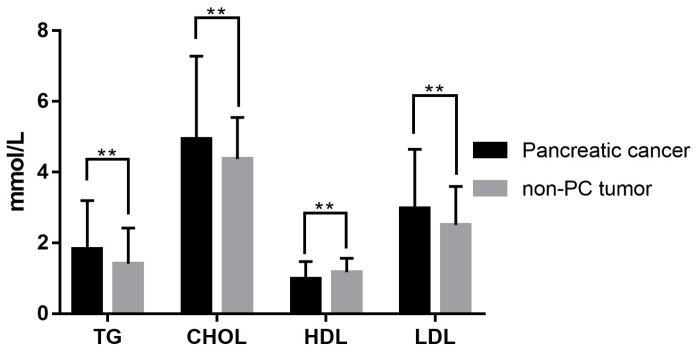
Serum lipid difference between PC and non-PC tumor patients in our cohort. **, *P*<0.05.

A total of 432 patients undergoing surgical resection at the two centers from January 2012 to December 2019 were included in this study. Among them, 260 patients were diagnosed as PC and 172 patients as non-PC tumors pathologically. Their demographic and clinical characteristics, as well as laboratory information, were summarized in Table 1. There were 167 males (67.5%; median age, 61 years; range, 23-94 years) in the PC group and 87 males (50.6%; median age, 55 years; range, 16-82 years) in the non-PC tumor group. Compared with non-PC tumor patients, PC patients showed significant differences in age (P<0.001), gender (P=0.005), and symptomatology (P<0.001) at baseline. Elder age, male gender, alcohol intake (P=0.011), and the first symptoms as abdominal pain and jaundice could be risk factors for PC. Among laboratory indicators, PC patients exhibited lower hemoglobin (P=0.002), lower albumin level (P<0.001) and higher levels of total bilirubin (P<0.001), alanine aminotransferase (P<0.001), serum glucose (P<0.001), and CA199 (P<0.001) in serum. Besides, activated partial thromboplastin times were significantly prolonged in the PC group (P=0.02) versus patients with non-PC tumors. As shown in Table 1 and Figure 2, PC patients presented a serum lipids difference from non-PC tumor patients in triglyceride (P=0.001), cholesterol (P=0.005), HDL (P<0.001), LDL (P=0.003), apolipoprotein A1 (P=0.001), apolipoprotein B (P=0.007), and apolipoprotein E (P<0.001).

### Logistic regression analysis of risk factors for PC

The univariable logistic regression analysis revealed that male gender (P=0.005), elder age (P=0.002), alcohol intake (P=0.012), lower hemoglobin (0.002), higher levels of total bilirubin (P<0.001), alanine aminotransferase (P<0.001), serum glucose (P=0.001), and CA199 (P<0.001), together with dyslipidemia (triglyceride: P=0.007, cholesterol: P=0.003, HDL: P=0.012, and LDL: P=0.015), were significantly correlated with PC. These characteristics above were further screened using the multivariable analysis. As shown in Table 2, only increased total bilirubin (P=0.004), alanine aminotransferase (P=0.002), and CA199 (P<0.001) levels on admission were the independent risk factors of PC.

### Clinicopathological features and survival analysis between PC patients with different lipid levels

**Figure 3:**
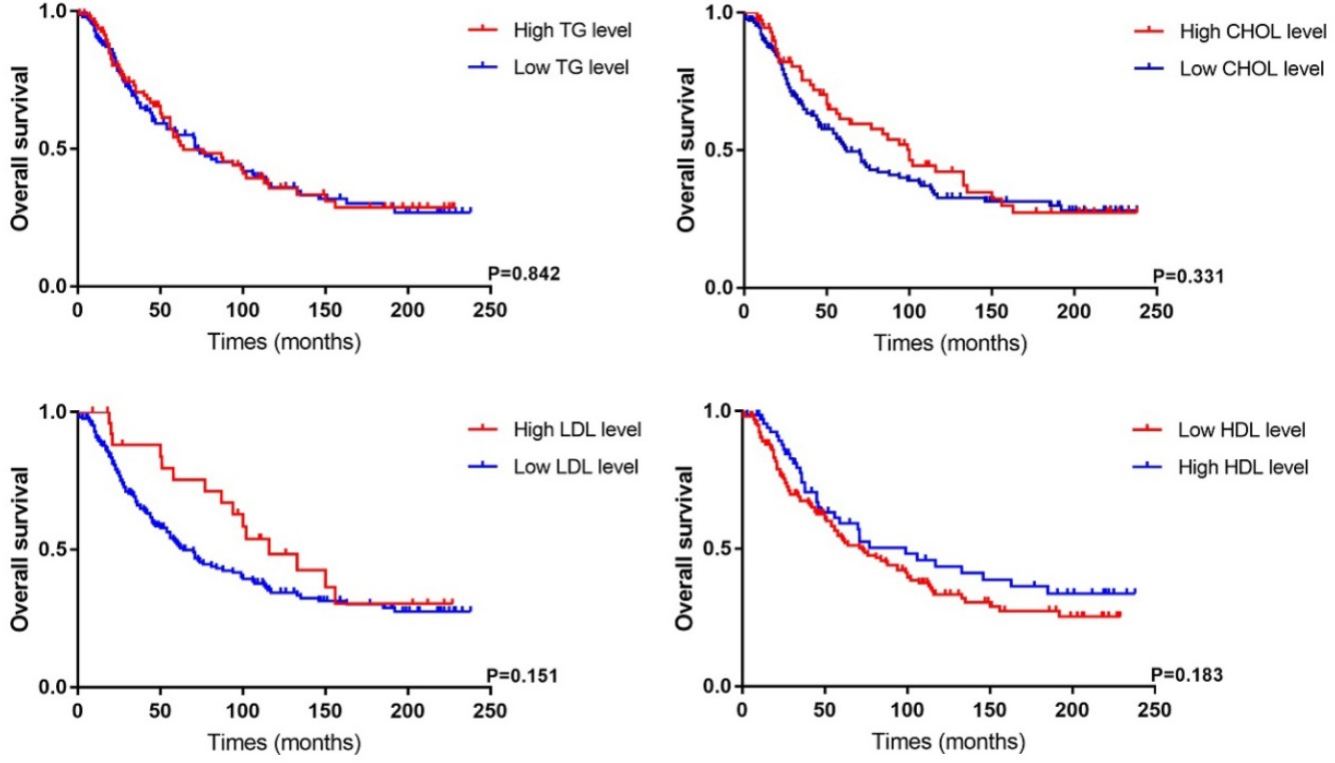
Survival analysis of PC patients according to different lipid level in our cohort.

As listed in Table 3, clinicopathological features and prognostic data of PC patients were analyzed based on different lipid level. We found cholesterol (P<0.001) and LDL (P<0.001) levels were related to tumor size. PC patients with a high LDL level had longer follow-up (P=0.009) because of the within-group difference in number of cases. According to an LDL cutoff of 4.14 mmol/L, 27 PC patients were assigned to the high-LDL group versus 201 patients in the low-LDL group. Besides, there were non-significant differences in other indicators, including tumor stage, degree of differentiation, nerve and vascular invasion, between the two groups. The Kaplan-Meier survival analysis showed that dyslipidemia in PC patients had no significant correlation with OS (Figure 3), while patients with a low HDL level tended to have a poor prognosis (P=0.183).

### Differential expression of LDLR and HDLBP mRNAs and their prognostic value in PC

**Figure 4:**
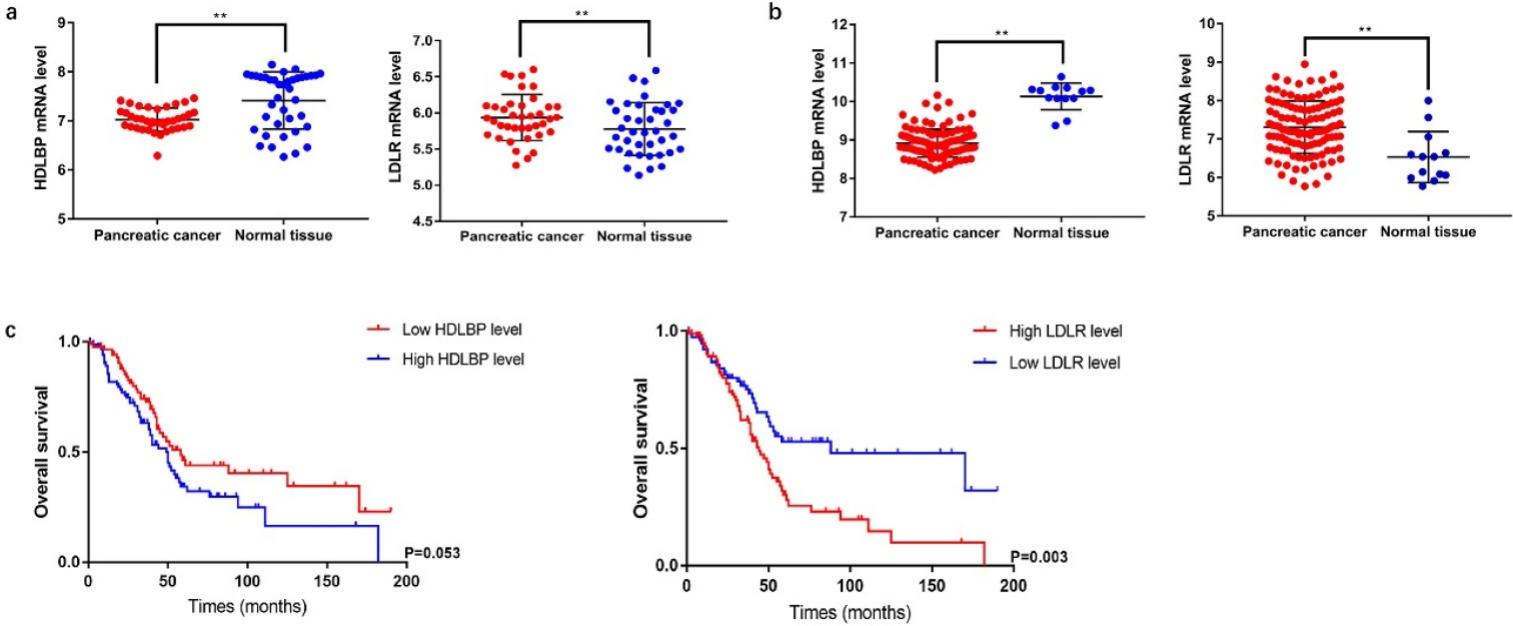
Differential and survival analysis of related lipoprotein from database data. **a** and** b,** The expression level of HDLBP and LDLR in PC tissue and paracancerous normal tissue from GSE15471 and GSE62165, ** P<0.05. **c** TCGA database was searched to analyze overall survival of PC patients in different HDLBP and LDLR expression level.

LDLR is a cell-surface receptor that metabolizes elevated levels of plasma LDL-cholesterol (LDL-C) to regulate cholesterol homeostasis in the circulation 7. HDLBP is a protein that specifically binds to HDL molecules and may function in the removal of excess cellular cholesterol 8. We analyzed the mRNA expression levels of LDLR and HDLBP using GSE15471 and GSE62165 cohorts from GEO. The results showed that LDLR (GSE15471: P=0.044; GSE62165: P<0.001) mRNA expression significantly increased in PC tissues, and HDLBP (GSE15471: P<0.001; GSE62165: P<0.001) mRNA expression were downregulated compared with the expressions in paired adjacent noncancerous tissues. Subsequently, differences in clinical features between patients with different LDLR and HDLBP mRNA expression levels were compared using the data from 177 PC patients in TCGA database. The basic characteristics and follow-up information were presented in Table S1. The results showed that PC patients with a high LDLR expression level were associated with a poor prognosis (P=0.003) (Figure 4), and patients with both high LDLR (P=0.001) and HDLBP (P=0.042) expressions exhibited a higher incidence of cancer-related death versus those with low LDLR and HDLBP expressions (Table S2).

## Discussion

Metabolic reprogramming has been ascertained to be a hallmark of cancer, of which abnormal glucose and lipid metabolism are key players promoting the progression of PC 5, 9. However, differences in baseline serum lipid levels between PC and non-PC tumor types are rarely noticed. Our results demonstrated these disparities in baseline clinical features and laboratory parameters between the two groups. Specifically, PC patients with dyslipidemia had higher serum triglyceride, cholesterol, and LDL levels and a decreased HDL level. Compared to common diagnostic factor for PC, CA199 for example, these lipid indicators did not show a notable role in diagnosis and prognosis evaluation of PC. Regarding lipid-related mRNA markers LDLR and HDLBP (the cellular receptors for LDL and HDL), they also displayed a potential diagnostic performance, showing differential expressions between PC and paracancerous tissues. And PC patients with a high LDLR level significantly correlated with a poor prognosis, as indicated by the TCGA survival analysis.

In the results of our study, lipid regulation in PC is more conducive to elevating serum lipid levels compared to other non-PC tumor types. Some pieces of verifiable evidence support that high dietary cholesterol is associated with an increased risk of PC 2-3. Food-derived fatty acids are required for the synthesis of phospholipids to in cell proliferation and lipid metabolism signaling 10-11. Because lipids such as phospholipid bilayers are fundamental structural components enabling cell division 6. So high serum fat levels contribute to PC development. Meanwhile, to adapt the nutritional needs increase to withstand hypoperfusion and hypoxia caused by high interstitial fluid pressures and poor stroma perfusion in the tumor environment, PC cells often manage to survive and develop by fueling lipid transport and metabolism 12-13. Besides, enzyme activities in lipid signaling are also strengthened. The expressions of lipogenic enzymes participating in the lipid metabolism, such as fatty acid synthase (FASN) and ATP citrate lyase (ACLY), are markedly upregulated in PC patients with poor survival by enhancing chemotherapy resistance 14-15. These findings suggested how PC cells use the regulation within the lipid microenvironment to promote PC progression. High lipid intake promotes pancreatic carcinogenesis, and PC cells induce cancer development by aberrant lipid metabolism activity.

LDLR is a transcriptional target of SREBP-2. It is also the LDL receptor that functions in increasing serum cholesterol levels in cells through receptor-mediated endocytosis 16. A higher LDLR expression level is associated with an increased risk of PC recurrence. After LDLR silencing, cancer cell proliferation can be significantly suppressed with enhanced gemcitabine sensitivity 15. Cholesterol uptake disruption via inactivating LDLR is warranted to develop a novel approach of PC metabolic targeting 9, 17. HDLBP, also known as vigilin, was found overexpressed in PC tissues in our study. However, evidence about the relationship between HDLBP and PC is limited. In other cancer types, hepatocellular carcinoma, for example, HDLBP is proved to act as an anti-apoptotic effect in promoting cell proliferation and tumor growth 18. Since we ascertained the downregulation of HDLBP mRNA expression in this work, whether HDLBP involves in PC onset and development needs further research.

New progress has confirmed cholesterol uptake and lipid metabolism as a highly attractive target for PC metabolic therapy. Statins, inhibitors of HMG-CoA reductase, have been studied *in vitro* using various cancer cell lines. Antitumor effects of lipophilic statins in PC result mainly from the suppression of proliferation and promotion of apoptosis 19-20. The roles of statins in improving PC survival also have been reported in some clinical trials 21-22. Orlistat, a FASN inhibitor, induces endoplasmic reticulum stress and increases gemcitabine sensitivity in mouse models with orthotopic PC implantation 23.

In summary, our study elaborated disparities in dyslipidemia between PC and non-PC tumor patients, but the underlying mechanism has not been fully explored. The roles of specific lipoproteins in PC and their diagnostic and prognostic values were verified. Moreover, key proteins involving lipid metabolic regulation, especially LDLR, are potential prognostic targets for PC. As well, lipid metabolic regulation provides a potential therapy for PC in clinical applications.

## Conclusion

This study highlights dyslipidemia in PC patients and its difference between PC and non-PC tumor types but differentially expressed lipid indicators exhibit no obvious correlations with PC prognosis. Among the related lipoprotein, LDLR is a potential predictive marker for PC prognosis. A new view to distinguish PC from other pancreatic tumors is raised by aberrant blood lipid levels. Our study underlies further PC research on lipid metabolism and the application of lipid-regulating drugs in PC therapy. There were still several limitations in this study. As a retrospective study, the dyslipidemia in PC requires to be investigated in a prospective study, and the mechanism of how dyslipidemia affects PC onset and progression requires validations. Negative results of these lipid indicators in diagnostic and survival analysis may be related to the small size and short follow-up periods. Roles of LDLR and HDLBP in PC need to be further verified in experimental research. Nevertheless, this two-center retrospective study, with certain reliability, shed new light on lipid metabolism in PC.

## Figures and Tables

**Table 1:** Demographic characteristics and laboratory data of patients with PC and non-PC tumor

Characteristics		Pancreatic cancer		non-PC tumor	P-value
Age, years	Σ260	60.48±10.77	Σ172	53.29±14.41	**<0.001**
Gender, male	Σ260	167 (64.2%)	Σ172	87 (50.6%)	**0.005**
Smoke	Σ260	62 (23.8%)	Σ172	34 (19.8%)	0.319
Alcohol	Σ260	51 (19.6%)	Σ172	18 (10.5%)	**0.011**
BMI index, kg/m^2^	Σ260	22.17±3.23	Σ172	22.32±3.03	0.639
**Comorbidities**					0.545
Circulatory system diseases	Σ260	69 (26.5%)	Σ172	41 (23.8%)	
Metablolic diseases	Σ260	53 (20.4%)	Σ172	24 (14.0%)	
Chornic inflammation	Σ260	18 (6.9%)	Σ172	17 (9.9%)	
Other malignant tumor	Σ260	8 (3.1%)	Σ172	4 (2.3%)	
**Symptom**					**<0.001**
Abdominal pain	Σ260	146 (56.2%)	Σ172	60 (34.9%)	
Jaundice	Σ260	95 (36.5%)	Σ172	8 (4.7%)	
Asymptomatic	Σ260	31 (11.9%)	Σ172	90 (52.3%)	
Others	Σ260	18 (6.9%)	Σ172	17 (9.9%)	
**Laboratory data**					
White cell count, ×10^9^/L	Σ255	5.62±1.62	Σ172	5.86±1.93	0.168
Hemoglobin, g/L	Σ255	123.97±17.20	Σ172	129.39±17.58	**0.002**
Platelet count, ×10^9^/L	Σ255	214.12±74.61	Σ172	208.09±75.13	0.414
Total bilirubin, μmol/L	Σ255	102.54±118.77	Σ171	22.38±49.9	**<0.001**
Alanine aminotransferase, U/L	Σ254	140.89±176.78	Σ171	42.90±85.03	**<0.001**
Albumin, g/L	Σ255	39.21±5.21	Σ170	41.05±5.24	**<0.001**
Creatinine, μmol/L	Σ255	62.38±15.50	Σ171	61.68±16.38	0.657
Serum glucose, mmol/L	Σ250	6.72±2.69	Σ165	5.61±1.90	**<0.001**
Prothrombin time, s	Σ252	12.38±1.54	Σ170	12.12±1.54	0.085
Activated partial thromboplastin time, s	Σ251	33.91±6.80	Σ169	32.36±6.41	**0.020**
CA199, U/ml	Σ238	450.23±493.76	Σ163	32.19±107.84	**<0.001**
**Serum lipids level**					
Triglyceride, mmol/L	Σ242	1.82±1.37	Σ157	1.41±1.01	**0.001**
Cholesterol, mmol/L	Σ242	4.93±2.35	Σ158	4.36±1.18	**0.005**
High density lipoprotein, mmol/L	Σ231	0.98±0.49	Σ147	1.17±0.39	**<0.001**
Low density lipoprotein, mmol/L	Σ229	2.97±1.67	Σ146	2.50±1.09	**0.003**
Lipoprotein a, mmol/L	Σ82	207.22±361.98	Σ80	264.99±444.73	0.365
Apolipoprotein A1, g/L	Σ82	1.11±0.38	Σ81	1.29±0.29	**0.001**
Apolipoprotein B, g/L	Σ82	0.88±0.35	Σ81	0.76±0.22	**0.007**
Apolipoprotein E, mg/L	Σ82	64.21±58.59	Σ81	35.62±23.89	**<0.001**

**Table 2:** Logistic regression analysis of risk factors for PC

	Univariable analysis		Multivariable analysis	
	Odd ratio (95% CI)	*P* value	Odd ratio (95% CI)	*P* value
Sex, male	0.57 (0.39, 0.84)	**0.005**		
Age, ≥60 years	1.89 (1.27, 2.82)	**0.002**		
Alcohol	2.09 (1.17, 3.72)	**0.012**		
Hemoglobin, <120 g/L	1.91 (1.23, 2.97)	**0.004**		
Total bilirubin, >17.1 μmol/L	8.30 (5.05, 13.64)	**<0.001**	2.88 (1.39, 5.97)	**0.004**
Alanine aminotransferase, >50 U/L	6.58 (4.21, 10.29)	**<0.001**	3.46 (1.55, 7.69)	**0.002**
Albumin, <35 g/L	1.27 (0.72, 2.24)	0.417		
Serum glucose, >7.0 mmol/L	2.45 (1.47, 4.08)	**0.001**		
Activated partial thromboplastin time, >40 s	1.21 (0.68, 2.14)	0.521		
CA199, >25 U/ml	18.78 (11.30, 31.22)	**<0.001**	10.87 (5.97, 19.80)	**<0.001**
Triglyceride, >1.7 mmol/L	1.82 (1.18, 2.81)	**0.007**		
Cholesterol, >5.18 mmol/L	2.10 (1.28, 3.45)	**0.003**		
High density lipoprotein, <1.16 mmol/L	1.74 (1.13, 2.67)	**0.012**		
Low density lipoprotein, >4.14 mmol/L	3.10 (1.25, 7.69)	**0.015**		

**Table 3:** Clinicopathological and follow-up information of PC patients in different lipid level

	High CHOL	Low CHOL	*P* value	High TG	Low TG	*P* value	Low HDL	High HDL	*P* value	High LDL	Low LDL	*P* value
Tumor size, cm	73	164	**<0.001**	96	141	0.057	158	69	0.869	27	201	**<0.001**
	2.91±1.04	3.63±1.48		3.20±1.24	3.55±1.47		3.37±1.41	3.40±1.38		3.50±1.40	2.55±1.00	
**Operation type**	73	169	0.143	97	145	0.102	162	69	0.124	27	202	0.218
Radical resection	71 (97.3%)	156 (92.3%)		94 (96.9%)	133 (91.7%)		152 (93.8%)	68 (98.6%)		27 (100.0%)	191 (94.6%)	
Palliative resection	2 (2.7%)	13 (7.7%)		3 (3.1%)	12 (8.3%)		10 (6.2%)	1 (1.4%)		0 (0.0%)	11 (5.4%)	
**AJCC stage**	73	169	0.055	97	145	0.074	162	69	0.246	27	202	0.961
Stage I, II	56 (76.7%)	113 (66.9%)		74 (76.3%)	95 (65.5%)		118 (72.8%)	45 (65.2%)		20 (74.1%)	141 (69.8%)	
Stage III, IV	17 (23.3%)	56 (33.1%)		23 (23.7%)	50 (34.5%)		44 (27.2%)	24 (34.8%)		7 (25.9%)	61 (30.2%)	
**Differentiation degree**	67	143	0.601	86	128	0.394	146	62	0.186	25	178	0.417
Well	13 (19.4%)	27 (18.9%)		15 (17.4%)	25 (19.5%)		27 (18.5%)	12 (19.4%)		7 (28.0%)	31 (17.4%)	
Moderate	36 (53.7%)	71 (49.7%)		41 (47.7%)	66 (51.6%)		68 (46.6%)	36 (58.1%)		11 (44.0%)	93 (52.2%)	
Poor	18 (26.9%)	45 (31.5%)		30 (34.9%)	37 (28.9%)		51 (34.9%)	14 (22.6%)		7 (28.0%)	54 (30.3%)	
**Nerve invasion**	67	148	0.574	89	126	0.664	142	66	0.977	26	180	0.269
	37 (55.2%)	80 (54.1%)		50 (56.2%)	67 (53.2%)		75 (52.8%)	35 (53.0%)		13 (50.0%)	96 (53.3%)	
**Vascular invasion**	68	148	0.874	90	126	0.977	143	66	0.140	26	181	0.753
	12 (17.6%)	31 (20.9%)		18 (20.0%)	25 (19.8%)		32 (22.4%)	9 (13.6%)		3 (11.5%)	37 (20.4%)	
**Follow-up, months**	73	169	0.083	97	145	0.846	162	69	0.065	27	202	**0.009**
	80.99±69.65	64.86±64.57		70.74±63.92	69.04±68.24		66.57±62.13	84.38±76.74		102.67±67.72	67.98±66.36	
**Disease related death**	73	169	0.959	97	145	0.774	162	69	0.397	27	202	0.991
	40 (54.8%)	92 (54.4%)		54 (55.7%)	78 (53.8%)		92 (56.8%)	35 (50.7%)		15 (55.6%)	112 (55.4%)	

## Abbreviations

PC: Pancreatic cancer
Pnet: Pancreatic neuroendocrine tumor
IPMN: Intraductal papillary mucinous neoplasms
CP: Chronic pancreatitis
SPT: Solid pseudopapillary tumor
TG: Triglyceride
CHOL: Cholesterol
HDL: High density lipoprotein
LDL: Low density lipoprotein
GEO: Gene Expression Omnibus
TCGA: The Cancer Genome Atlas
HDLBP: High density lipoprotein binding protein
LDLR: Low density lipoprotein receptor
CA199: Caner antigen 199
AJCC: American Joint Committee on Cancer
OS: Overall survival
