# Supplementary material for: Dyslipidemia in Chinese Pancreatic Cancer Patients: A Two-Center Retrospective Study
Source: J Cancer. 2021 Jul 3;12(17):5338–44. doi: 10.7150/jca.60340 (PMC8317532; doi:10.7150/jca.60340)
Supplement: Supplementary file 1 — Supplementary tables. [file jcav12p5338s1.pdf]

Table S1. Clinicopathological and follow up information of PC patients in TCGA database.

No	177
Age, years	65.29±10.84
Gender, male	97 (54.8%)
Race	
White	156 (90.2%)
Asia	11 (6.4%)
Black	6 (3.5%)
Lipoprotein mRNA level	
LDLR	12.89±1.15
HDLBP	14.94±0.49
Tumor location, head	129 (80.6%)
AJCC stage	
stage I、II	167 (96.0%)
stage III、IV	7 (4.0%)
Follow-up, months	45.72±36.86
Disease related death	92 (52.6%)

Table S2. Clinicopathological and follow up information of PC patients in different lipoprotein expression level from TCGA database.

	High LDLR	Low LDLR	P value	High HDLBP	Low HDLBP	P value
Age, years	96	81	0.364	88	89	0.109
	65.97±11.52	64.48±9.98		66.60±11.14	63.99±10.43	
Gender, male	96	81	0.275	88	89	0.082
	49(51.0%)	48(59.3%)		54(61.4%)	43(48.3%)	

Race	92	81	0.644	87	86	0.477
White	82(89.1%)	74(91.4%)		77(88.5%)	79(91.9%)	
Asia	7(7.6%)	4(4.9%)		7(8.0%)	4(4.7%)	
Black	3(3.3%)	3(3.7%)		3(3.4%)	3(3.5%)	
AJCC stage	96	78	0.915	87	87	0.054
stage I、II	92(95.8%)	75(96.2%)		81(93.1%)	86(98.9%)	
stage III、IV	4(4.2%)	3(3.8%)		6(6.9%)	1(1.1%)	
Follow-up, months	96	79	0.088	88	87	0.225
	41.41±31.61	50.96±42.00		42.35±33.05	49.13±40.26	
Disease related death	96	79	**0.001**	88	87	**0.042**
	61(63.5%)	31(39.2%)		53(60.2%)	39(44.8%)	
